# Feed intake and efficiency of grazing Holstein-Friesian dairy cows divergent in genetic merit for live weight or milk production

**DOI:** 10.3168/jdsc.2026-1044

**Published:** 2026-07-06

**Authors:** M. Williams, D.P. Berry

**Affiliations:** Animal and Grassland Research and Innovation Centre, Teagasc, Moorepark, Fermoy, Co. Cork, P61 P302, Ireland

## Abstract

Accurately measuring individual animal DMI in grazing dairy cows remains expensive, invasive, and time-consuming, making the inclusion of direct measures of feed intake or efficiency in genetic evaluations for pasture-based systems challenging. Consequently, live weight is commonly used as an indicator of feed requirements in dairy cow total merit indexes, with the economic value for feed intake often applied directly to an animal's genetic merit for live weight. However, the biological appropriateness of this strategy at a population level has not been evaluated using large-scale intake data. The objective of the present study was to quantify the mean phenotypic net energy intake (NEI) and energy efficiency across lactation of grazing Holstein-Friesian cows stratified into deciles based on genetic merit for live weight, testing the hypothesis that energy intake, on average, increases with increasing genetic merit for live weight; stratification on genetic merit for fat- and protein-corrected milk was also explored. After edits, 3,163 DMI records from 2,201 lactations on 1,220 cows were available in early lactation, with corresponding mid- and late-lactation datasets comprising 3,703 records from 2,500 lactations and 2,758 records from 2,234 lactations, respectively. Within each stage of lactation, cows were stratified into 10 strata within their experimental treatment by year contemporary group based on their national paternal average EBV for live weight and separately on their EBV for fat- and protein-corrected milk; the mean phenotypic difference between extreme deciles was 60 kg (equivalent to 1.4 genetic SD) for live weight and 274 kg (equivalent to 0.88 genetic SD) for fat- and protein-corrected milk. The predicted marginal means for NEI and NEI adjusted for energy sinks in each of the EBV strata were estimated using linear mixed models. Across all stages of lactation, average NEI increased approximately linearly with increasing genetic merit for live weight, with mean differences of 0.9, 1.0, and 1.1 unité fourragère du lait (UFL) between cows in the extreme deciles in early, mid, and late lactation, respectively; regression of NEI means on stratum mean live weight EBV confirmed a strong linear association (R^2^ = 0.89, 0.90, and 0.80). When live weight EBV was fitted as a continuous covariate, individual cow NEI increased, on average, by 0.11, 0.08, and 0.10 (SE ranged from 0.02 to 0.03) UFL per 10-kg increase in live weight EBV in early, mid, and late lactation, respectively. In contrast, mean NEI adjusted for energy sinks declined with increasing genetic merit for live weight, with genetically heavier cows consuming, on average, 0.8, 0.9, and 0.6 UFL less net energy than genetically lighter cows in early, mid, and late lactation, respectively. When stratified on their genetic merit for fat- and protein-corrected milk, no stratum differences in mean NEI were observed in early lactation, whereas in mid and late lactation, cows of higher genetic merit for fat- and protein-corrected milk consumed, on average, more net energy than contemporaries that were genetically inferior for fat- and protein-corrected milk. Collectively, these results demonstrate a strong and largely linear relationship between genetic merit for live weight and phenotypic energy intake at the subpopulation level in grazing dairy cows, supporting the continued use of live weight EBV as tool to shift mean (sub)population feed intake in pasture-based breeding objectives.

## Graphical Abstract

**Figure:**
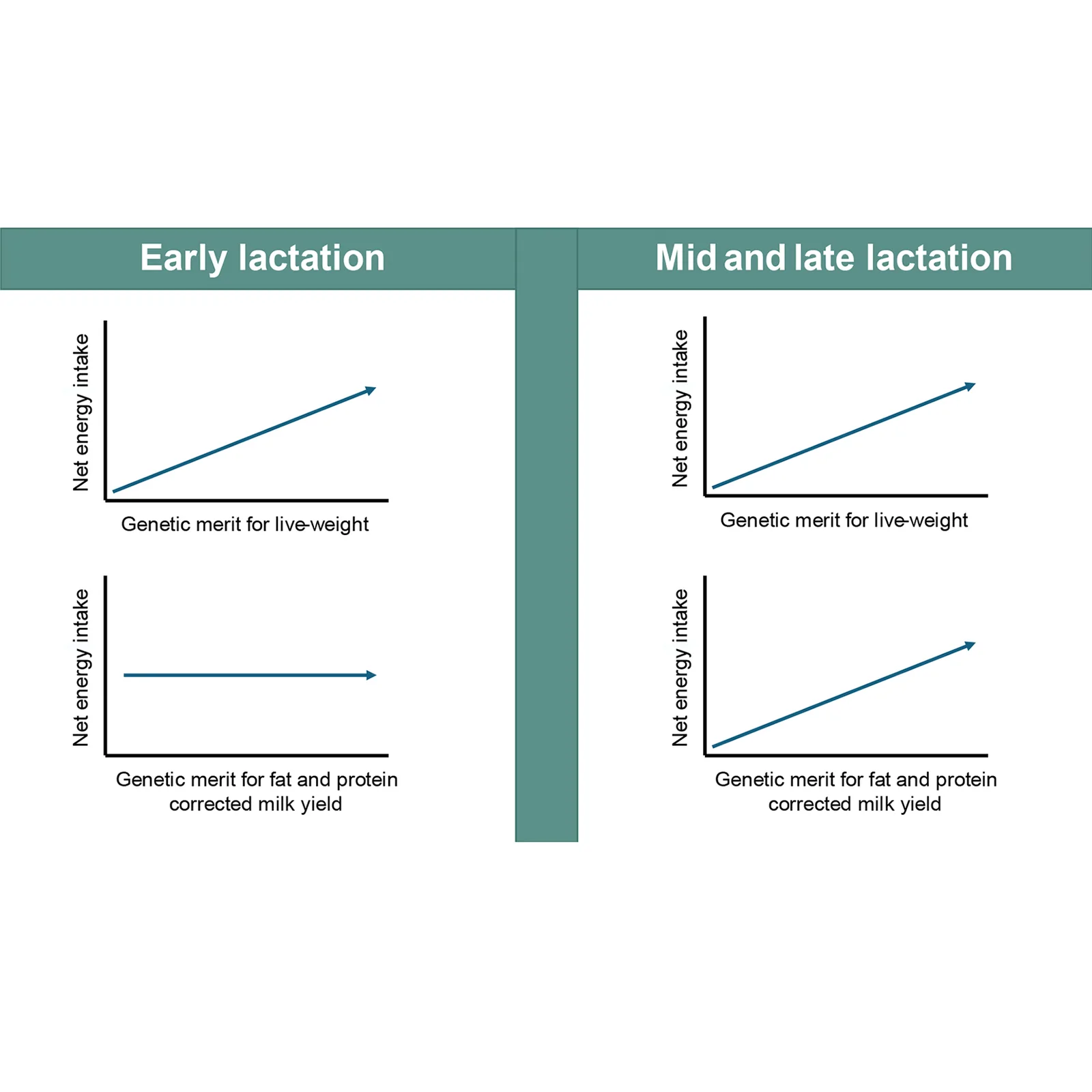

Accurately measuring individual feed intake in grazing dairy cows using the gold-standard n-alkane technique is invasive, expensive, and time-consuming (Williams et al., 2022). Despite a range of studies proposing alternative approaches to predict feed intake in dairy cows (Tran et al., 2010; McParland et al., 2014; Halachmi et al., 2016), pasture-based dairy systems continue to lack cost-effective and scalable methods for measuring individual animal feed intake. Consequently, large datasets of direct measurements of feed intake remain limited in grazing systems. Although several countries with predominantly confinement-based dairy production systems have incorporated explicit feed intake or feed efficiency traits into their national dairy breeding objectives (Pryce et al., 2015; CRV, 2022; AHDB, 2023; Stephansen et al., 2025), countries with predominantly pasture-based dairy production systems, such as Ireland and New Zealand, continue to use live weight as a proxy trait for feed intake (Berry et al., 2007; DairyNZ, 2024; ICBF, 2025). Live weight is already integrated into national genetic evaluation systems, making it an attractive predictor of feed intake in the absence of direct phenotypes, at least at the population level.

Positive phenotypic and genetic correlations between live weight and feed intake have been widely reported in both pasture-based (Williams et al., 2022) and confinement systems (Veerkamp and Brotherstone, 1997; Manafiazar et al., 2016; Manzanilla-Pech et al., 2016). Nevertheless, it remains unclear whether the relationship between genetic merit for live weight and actual feed intake is strictly linear. This question is important because if the relationship between genetic merit for live weight and phenotypic feed intake is not linear, the economic values applied to live weight in breeding objectives may not accurately reflect true average feed requirements. Such misrepresentation could result in biased predictions of feed costs and inaccurate rankings of animals on profitability.

Therefore, quantifying the relationship between genetic merit for live weight and phenotypic energy intake is necessary to ensure that the relationship between live weight EBV and phenotypic feed intake at a population level is biologically consistent in pasture-based production systems. The primary objective of the present study was to quantify the mean phenotypic net energy intake (**NEI**) and energy efficiency across lactation in grazing Holstein-Friesian cows stratified into deciles on genetic merit for live weight, testing the hypothesis that mean population level energy intake increases with increasing genetic merit for live weight. A secondary objective was to characterize the mean population level energy intake and efficiency in grazing dairy cows stratified on genetic merit for milk production.

Records of individual cow daily grass DMI, concentrate DMI, milk production, live weight, and BCS were available from the Animal and Grassland Research and Innovation Centre (Teagasc Moorepark, Co. Cork, Ireland). The data originated from 5 separate research farms and were collected between the years of 1998 and 2022, inclusive. The DMI records were collected as part of experimental protocols evaluating different genotypes of Holstein-Friesian dairy cows (≥87.5% Holstein-Friesian) and alternative grazing and nutritional strategies. All cows grazed freely under a rotational grazing system and grazed predominantly on perennial ryegrass (*Lolium perenne.* spp.) swards. Each cow received between 0 and 6.2 kg DM of concentrate per day. The grass DMI of all animals was estimated using the n-alkane technique described by Mayes et al. (1986) and modified by Dillon and Stakelum (1989); the DMI estimation was repeated an average of 5.6 times per cow and repeated an average 2.7 times per cow within each lactation. To estimate daily grass DMI of an individual cow, all cows were dosed twice daily with dotriacontane for 12 d, with forage and fecal samples collected for 6 d from the seventh day of dosing. Following the dosing and the collection period, fecal samples were bulked by cow, dried, and the alkane content quantified.

Individual cow milk yield was recorded daily using electronic milk meters (Dairymaster, Causeway, Co. Kerry, Ireland), with milk fat and protein concentration determined weekly from successive morning and evening milk samples using mid-infrared spectroscopy (Fos-let instrument, AS/N Foss Electric, Hillerød, Denmark). Cow live weight was recorded using an electronic weighing scale (Tru-Test Limited, 25 Carbine Road Mount Wellington, Auckland, New Zealand) every 1 to 3 wk, and cow BCS was recorded on a scale of 1 to 5 in increments of 0.25 (Edmundson et al., 1989) approximately every 3 wk. Cubic splines with 6 knot points at 20, 70, 120, 220, and 270 DIM, with a covariance structure fitted among the knot points, were fitted through the individual live weight and BCS test-day records as described by McParland et al. (2014). Live weight and BCS at each DIM were interpolated from the fitted splines, and forward differencing was used to estimate daily live weight and BCS change for each DIM.

Before editing, 11,993 individual animal DMI records were available from 4,067 lactations on 1,855 Holstein-Friesian dairy cows. Individual DMI, milk production, live weight, and BCS estimates were removed if they were more than 3 SD from the parity by stage of lactation mean. Only the milk yield and milk composition record closest to the DMI measurement was retained and, if no milk yield measurement was recorded within 7 d of the DMI measurement, the DMI measurement was filtered out. Records from cows without a recorded sire were discarded. Cow parity (ranging from 1 to 10) was categorized as 1, 2, 3, 4, or ≥5. Days in milk were categorized into approximately 30-d intervals, and the records were divided into early (≤90 DIM), mid (91–180 DIM), and late lactation (>180 DIM). Contemporary group was defined as experimental treatment by year and contemporary groups, with fewer than 5 DMI records discarded. After editing, the early lactation dataset comprised 3,163 DMI records from 2,201 lactations on 1,220 Holstein-Friesian cows, the mid lactation dataset comprised 3,703 DMI records from 2,500 lactations on 1,368 Holstein-Friesian cows, and the late lactation dataset comprised 2,758 DMI records from 2,234 lactations on 1,175 Holstein-Friesian cows.

Net energy intake was calculated as

NEI = NE − ΔNE,

where

NE = (grass DMI × pasture energy value) + (concentrate DMI × concentrate energy value),

ΔNE = 6.3 × PrC^2^ + 0.002 × NE^2^ − 0.017 × NE,

and where NE is the theoretical net energy intake (unité fourragère du lait [**UFL**]), ΔNE is the adjustment for the proportion of concentrate in the diet (UFL), and PrC is the proportion of concentrate in the diet. The energy values of concentrate and pasture were based on the French net energy system, where 1 UFL is the energy required for lactation equivalent to 1 kg of standard air-dry barley (Jarrige et al., 1986). The UFL of the concentrate was calculated from the chemical composition of the feed and the UFL of the herbage was calculated using the CP and ADF concentrations (Jarrige et al., 1986). Where the UFL of the offered herbage was not available, the month-year average was assumed, and where no month-year average was available, the month average was assumed. In addition to NEI, an efficiency trait was defined as the residual from a linear regression of NEI on energy sinks and other energy sources (**NEI_adj_**), as follows:

$$\text{\textbackslash{}[}\begin{array}{c} NEI_{adj}=NEI\;-[b_{0}+b_{1}NEL+b_{2}BW^{0.75}+b_{3}BCS+b_{4}\Delta BW^{+}\\ \;+b_{5}\Delta BW^{-}+b_{6}\Delta BCS^{+}+b_{7}\Delta BCS^{-}], \end{array}\text{\textbackslash{}]}$$

where *b*_0_ represents the intercept and *b*_1_ to *b*_7_ represent the respective partial regression coefficients of NEI on the respective energy sinks, NEI is net energy intake, NEL is the net energy requirement for lactation, and BW^0.75^ is metabolic live weight. The energy required for a 1-kg gain in live weight is higher than the energy generated from a 1-kg loss in live weight (O'Mara, 1996); therefore, piecewise regression was applied to BCS and live weight in the NEI_adj_ model, where ΔBW^+^ describes a cow gaining live weight and ΔBW^−^ describes a cow losing live weight. Similarly, ΔBCS^+^ describes a cow gaining BCS and ΔBCS^−^ describes a cow losing BCS. Fat- and protein-corrected milk (**FPCM**) yield (kg) to a standard of 4.0% fat and 3.3% protein was calculated as follows:

FPCM kg = (0.337 × Milk kg) + (11.6 × Fat kg) + (5.999 × Protein kg),

where Milk kg, Fat kg, and Protein kg are the daily milk, fat, and protein yields measured closest to the DMI estimates.

Paternal average EBV for live weight, milk yield, fat yield, and protein yield were obtained for all cows from the September 2025 Irish national genetic evaluation, and an EBV for FPCM was calculated. Within each stage of lactation, cows were stratified within their experimental treatment by year contemporary group into 10 strata based on their paternal average EBV for live weight and separately their parental average EBV for FPCM; the number of animals per EBV stratum was broadly balanced, ranging from 222 to 357, 269 to 427, and 201 to 319 across strata in early, mid, and late lactation, respectively, for live weight EBV, with similar distributions observed for FPCM EBV strata. The EBV for each trait was standardized and a variable (to be later used as a covariate in the statistical model) representing 10 equally spaced points across −2 to +2 SD was derived to impose equal distance in mean EBV between each of the adjacent strata. The inclusion of this term as a covariate in the statistical model facilitated modeling of linear differences in performance across the different EBV strata. Predicted marginal means for NEI and NEI_adj_ were estimated using linear mixed models in PROC MIXED in SAS 9.4 (SAS Institute Inc.) for each EBV stratum as

$$\text{\textbackslash{}[}\begin{array}{c} y_{ijklmn}=Parity_{j}\times DIM_{k}+EBV\;stratum_{l}+EBVcov\\ \;+CG_{m}+cow\_lact_{n}+e_{jklmno}, \end{array}\text{\textbackslash{}]}$$

where *y_ijklmn_* was the observed value of live weight or FPCM for cow *i*; *Parity_j_* was the fixed effect for parity *j* (*j* = 1, 2, 3, 4, ≥5); *DIM_k_* was the fixed effect for DIM class *k* (*k* = 1 to 10); *EBV stratum_l_* was the fixed effect for live weight or FPCM EBV stratum *l*; *EBVcov* was the continuous EBV covariate; *CG_m_* was the random effect of experimental treatment by year contemporary group *m* nested within farm; *cow_lact_n_* was the random effect of cow-lactation *n*, where *cow_lact_o_* $\&sim;\;N\left(0,I\sigma_{cow\_lact}^{2}\right)$ represents the within-lactation cow effects and **I** is the identity matrix; and *e_ijklmno_* was the residual term, where $e\&sim;N\left(0,I\sigma_{e}^{2}\right),$ $\sigma_{e}^{2}$ represents the residual variance, and **I** is the identity matrix.

To assess whether observed differences in NEI across live weight EBV strata were independent of milk production, a separate series of models was fitted in which a continuous variable representing either the EBV for FPCM or phenotypic FPCM was included as an additional covariate. Similarly, to evaluate whether differences in NEI across live weight EBV strata were independent of differences in phenotypic live weight, phenotypic live weight was included as a covariate in a linear mixed model along with fixed effects of DIM class, parity, the interaction between parity and DIM class, EBV stratum for live weight, and the EBV covariate; the fitted random effects were the same as all previous models. Finally, to quantify the expected change in NEI per unit increase in genetic merit, EBV for live weight or EBV for FPCM were each fitted separately as continuous covariates, in place of the EBV strata and the EBV covariate.

The predicted marginal means for NEI by EBV stratum for live weight and FPCM are presented in Figure 1, Figure 2, respectively. Mean daily NEI was 17.0 UFL (SD = 3.7 UFL) in early lactation, 17.8 UFL (SD = 3.4 UFL) in mid lactation, and 16.7 UFL (SD = 3.0 UFL) in late lactation. Mean daily FPCM was 27.0 kg (SD = 5.7 kg) in early lactation, 22.0 kg (SD = 4.8 kg) in mid lactation, and 17.4 kg (SD = 4.3 kg) in late lactation. The SD of the EBV for live weight of all animals in the September 2025 Irish national genetic evaluation was 43 kg; this signifies that of the animals used in this study, the mean difference in EBV between the extreme strata for live weight in early, mid, and late lactation was between 1.36 and 1.41 genetic standard deviations.

**Figure 1:**
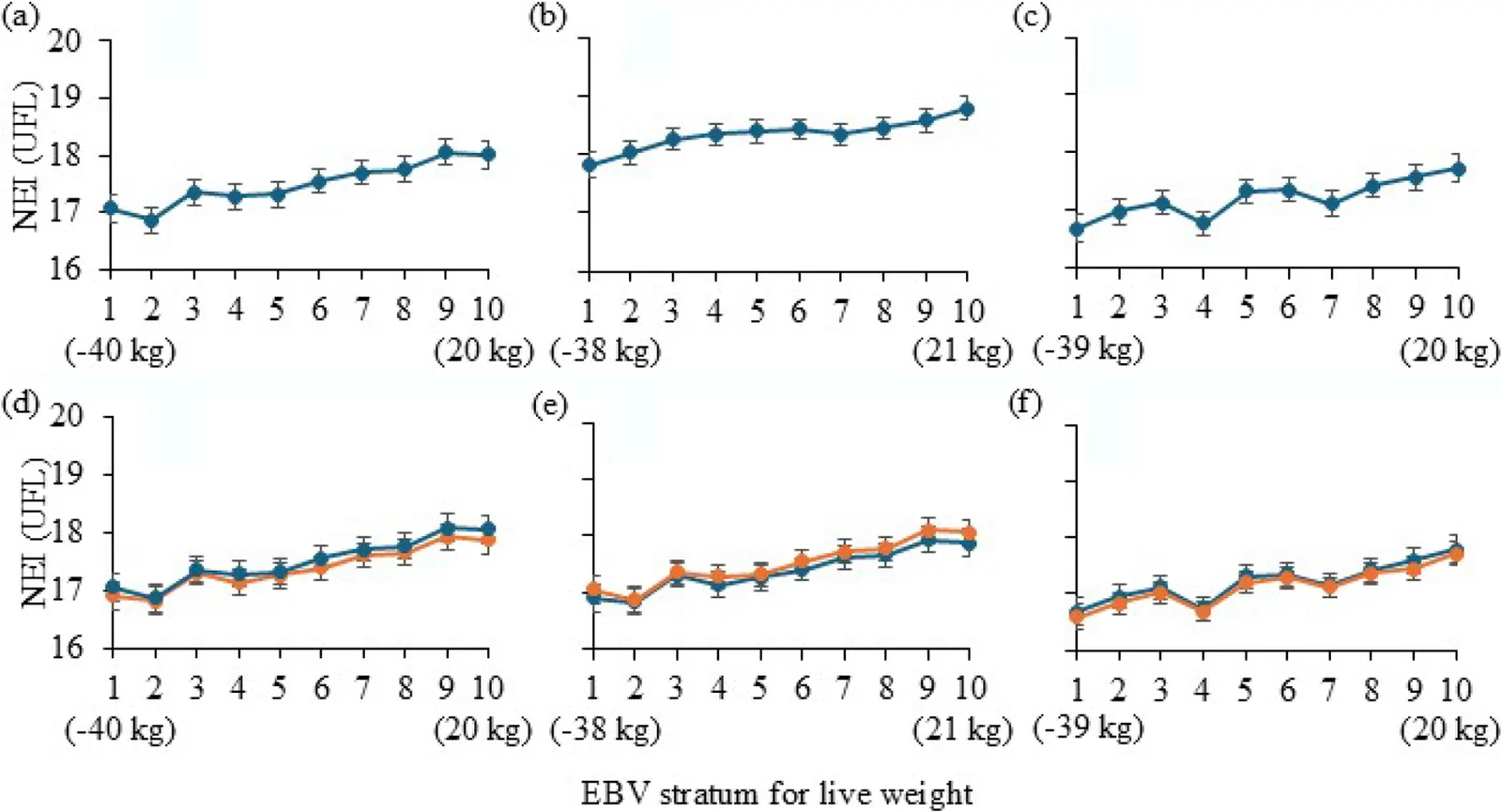
Predicted marginal means for net energy intake (NEI) in (a) early, (b) mid, and (c) late lactation when cows were stratified on EBV for live weight; and predicted marginal means for NEI adjusted for EBV for FPCM (blue) and phenotypic FPCM yield (orange) in (d) early, (e) mid, and (f) late lactation when cows were stratified on EBV for live weight. The mean EBV of the extreme strata for live weight are included in brackets. Standard error bars represent one SE above and below the predicted marginal mean.

**Figure 2:**
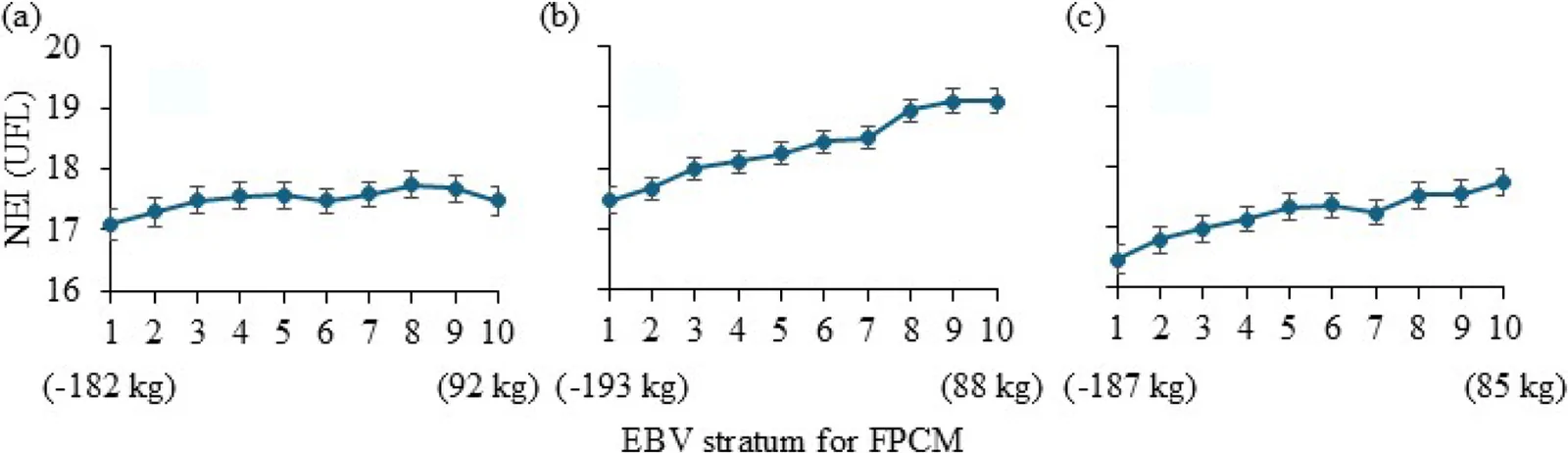
Predicted marginal means for net energy intake (NEI) in (a) early, (b) mid, and (c) late lactation when cows were stratified on EBV for fat- and protein-corrected milk (FPCM). The mean EBV of the extreme strata for FPCM are included in brackets. Standard error bars represent one SE above and below the predicted marginal mean.

Across all stages of lactation, mean NEI increased in an approximately linear manner with increasing genetic merit for live weight (Figure 1; *P* < 0.05). In early lactation, cows that were genetically the heaviest (i.e., highest live weight EBV stratum) consumed, on average, 0.9 UFL (standard error of the difference [**SED**] = 0.35) more NEI per day than the genetically lightest cows. In mid and late lactation, the genetically heaviest cows consumed, on average, 1.0 (SED = 0.30) and 1.1 UFL (SED = 0.34) more, respectively, than the genetically lightest cows. A simple linear regression fitted to the predicted mean NEI per stratum against the mean live weight EBV of each stratum indicated a strong linear relationship, with an R^2^ of 0.89, 0.90, and 0.80 in early, mid, and late lactation, respectively. When EBV for live weight was fitted as a continuous covariate in place of EBV strata, NEI increased, on average, by 0.11 UFL (SE = 0.03 UFL; *P* < 0.001) per 10-kg increase in live weight EBV in early lactation; corresponding increases in NEI of 0.08 (SE = 0.02 UFL; *P* < 0.001) and 0.10 UFL (SE = 0.02 UFL; *P* < 0.001) per 10-kg increase in the EBV for live weight were observed in mid and late lactation, respectively.

Including either EBV for FPCM or phenotypic FPCM as covariates when predicting the marginal means for NEI across live weight EBV strata did not materially alter the magnitude or direction of the relationship, indicating that the relationship between NEI and genetic merit for live weight at a group level was not conditional on either phenotypic or genetic merit for milk production (Figure 1). When phenotypic live weight was included as a covariate in the models, the association between genetic merit for live weight and NEI did change, although EBV stratum was still associated (*P* < 0.05) with mean NEI in mid and late lactation. However, in early lactation, there was no mean difference in NEI across live weight EBV strata after adjustment for phenotypic live weight. In contrast, in mid and late lactation, the genetically heaviest cows consumed, on average, less NEI than cows in the lightest EBV stratum, with mean differences in NEI between extreme live weight EBV strata of 0.84 UFL (SED = 0.30) in mid lactation and 0.61 UFL (SED = 0.30) in late lactation when phenotypic live weight was included as a covariate. This all implies that there is a genetic propensity, at a population level, for live weight to associate with NEI in mid to late lactation over and above the actual phenotypic live weight of the cow. This could point to the fact that genetically lighter cows have greater inherent maintenance requirements not fully captured by phenotypic live weight alone, which may be due to possible differences in metabolism or tissue composition, or indeed genetic effects on appetite regulation or intake capacity. In early lactation, however, the constraints on feed availability, and thus feed intake, mean that intake is, on average, determined more by factors other than genetic merit for live weight, such as the demands of lactation.

When cows were stratified on their genetic merit for FPCM, no mean difference in NEI was detected among EBV strata in early lactation (Figure 2; *P* = 0.37); this was despite there being a mean difference in the EBV for 305-d FPCM between the most extreme deciles of 274 kg, representing 0.9 of a genetic SD. In direct contrast, however, mid- and late-lactation cows genetically elite for FPCM consumed, on average, more NEI than cows that were genetically inferior for FPCM production, with mean differences of 1.6 (SED = 0.29) and 1.3 UFL (SED = 0.33), respectively, between the extreme strata. When EBV for FPCM was fitted as a continuous covariate in place of EBV strata, NEI increased, on average, by 0.2 UFL per day (SE = 0.06 UFL; *P* = 0.001) per 100-kg increase in 305-d FPCM EBV in early lactation; corresponding increases in daily NEI of 0.5 (SE = 0.05 UFL; *P* < 0.001) and 0.4 UFL (SE = 0.05 UFL; *P* < 0.001) per 100-kg increase in the EBV for 305-d FPCM were observed in mid and late lactation, respectively.

The predicted marginal means of NEI_adj_ in early, mid, and late lactation by EBV stratum for live weight are shown in Figure 3; mean NEI_adj_ decreased in a relatively linear manner with increasing genetic merit for live weight (*P* < 0.04), although this likely reflects the relatively narrow range in live weight, with any underlying nonlinear relationship in maintenance requirements not evident. On average, cows genetically expected to be the heaviest ate 0.8, 0.9, and 0.6 UFL less (SED ranged from 0.24 to 0.29) than expected relative to their genetically lightest contemporaries in early, mid, and late lactation, respectively. When stratified on their EBV for FPCM, there were no mean differences in NEI_adj_ in either early or late lactation, whereas cows genetically expected to produce more FPCM consumed, on average, more than expected in mid lactation relative to their contemporaries of lower genetic merit for FPCM (Figure 2).

**Figure 3:**
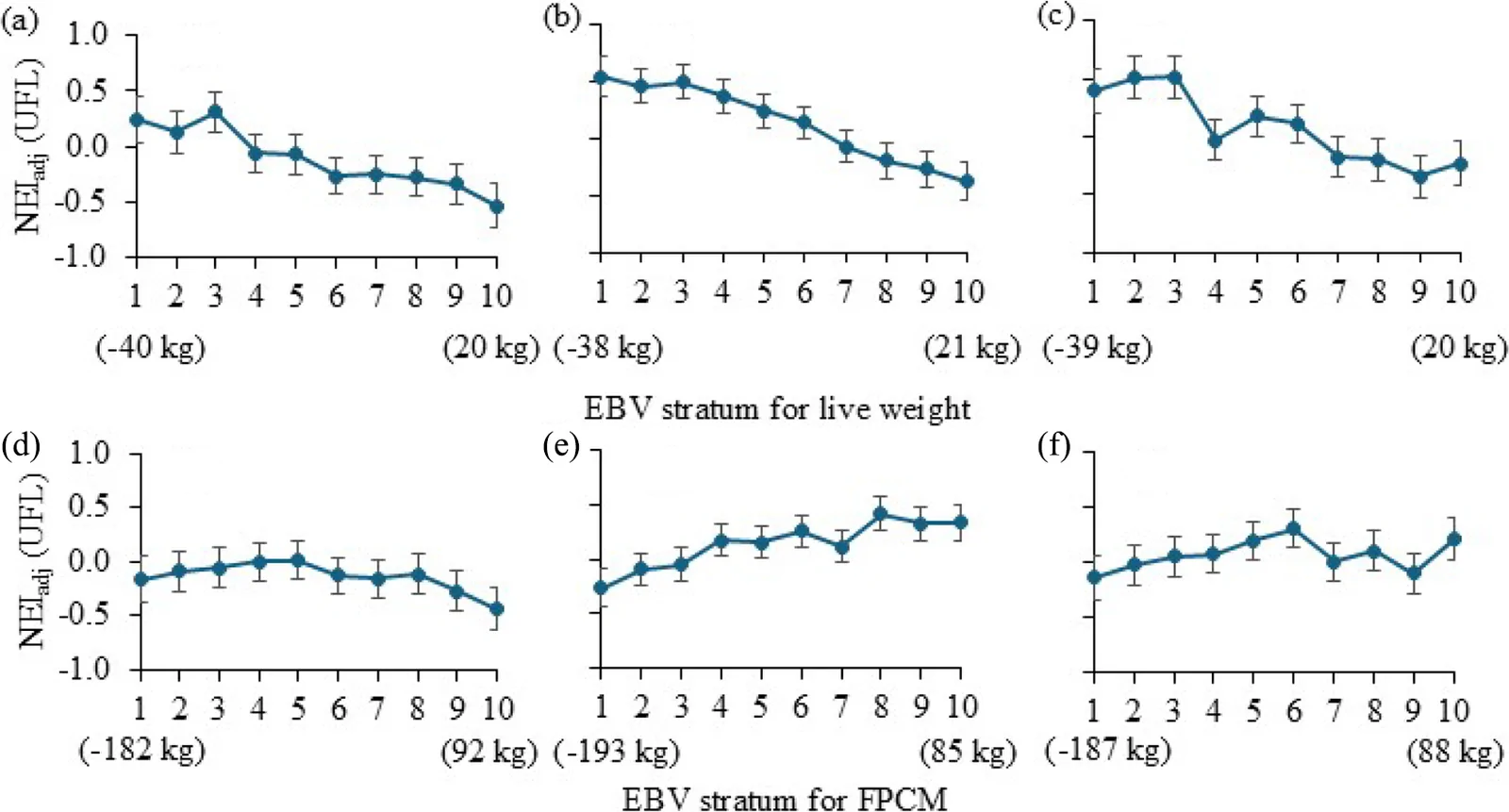
Predicted marginal means for net energy intake adjusted for energy sinks (NEI_adj_) in (a) early, (b) mid, and (c) late lactation when cows were stratified on their genetic merit (EBV) for live weight; and predicted marginal means for NEI_adj_ in (d) early, (e) mid, and (f) late lactation when cows were stratified on their EBV for energy corrected milk (FPCM). The mean EBV of the extreme strata for live weight and FPCM are included in brackets.

In pasture-based dairy production systems, where large-scale phenotyping of individual cow feed intake remains logistically challenging, genetic merit for live weight is routinely used as an indication of maintenance energy requirements in dairy cow total merit indexes. Implicit in this approach is the assumption that genetic differences in live weight are associated with predictable linear differences in actual energy intake, at least at a (sub-)population level. The strong linear increase in mean stratum NEI with increasing genetic merit for live weight observed across all stages of lactation in the present study indicates that this is true at a population level, at least for grazing Holstein-Friesian cows. Nonetheless, the linear relationship between NEI and genetic merit for live weight aligns with the established biological relationships between live weight and maintenance energy requirements (O'Mara, 1996) and with previous studies linking phenotypic live weight and phenotype feed intake in grazing dairy cow systems (Walsh et al., 2008; Williams et al., 2022). In fact, when the energy requirement for maintenance was calculated using the equation proposed by Jarrige (1989) and modified for Irish dairy systems by O'Mara (1996), the expected increase in energy required per 10-kg increase in live weight was 0.072 UFL. This theoretical expectation is broadly consistent with the regression coefficients observed in the present study when EBV for live weight was fitted as a continuous covariate; specifically, NEI increased by, on average, 0.11, 0.08, and 0.10 UFL per 10-kg increase in live weight EBV in early, mid, and late lactation, respectively. Furthermore, the persistence in the association between NEI and live weight EBV after accounting for either genetic merit or phenotypic milk production indicates that the increase in NEI reflects an underlying relationship between genetic merit for live weight and maintenance-related energy demand rather than being mediated through differences in production level.

Nevertheless, when cows were compared at a common phenotypic live weight, genetically heavier cows consumed, on average, less NEI than genetically lighter cows in mid and late lactation, which suggests that genetically heavier cows may have lower inherent energy requirements not fully captured by phenotypic live weight. These differences were not due to differences in BCS or BCS change because mean NEI_adj_ also declined with increasing genetic merit for live weight, such that genetically heavier cows, on average, still ate less than expected relative to genetically lighter cows. Consequently, the lower energy requirements of genetically heavier cows may reflect differences in digestive efficiency or physical activity levels between genetically heavier and lighter cows. Nevertheless, care should be taken when considering NEI_adj_ as a metric for efficiency in lactating cows, as NEI_adj_ is derived as a residual after adjusting for expected energy sinks; therefore, heavier cows may appear to be more efficient on a residual basis despite partitioning proportionally more energy toward maintenance (Coleman et al., 2010).

The absence of any association between EBV for FPCM and NEI during early lactation when cows were stratified on their genetic merit for FPCM suggests that cows genetically elite for milk production are constrained in their ability to increase intake in early lactation; consequently, cows genetically expected to produce more FPCM are likely relying on body reserves in early lactation to support production, consistent with previous observations in grazing cows (Walsh et al., 2008). Nevertheless, the expected reliance on body reserves in early lactation was not reflected in a greater loss of BCS or live weight in the present study, suggesting that BCS change alone may not capture the full variation in tissue mobilization or metabolic efficiency in early lactation. In mid and late lactation, when constraints on intake capacity relative to production are likely relaxed in grazing production systems, the expected positive association between FPCM EBV and NEI was more evident. This relationship between genetic merit for FPCM and NEI reinforces the importance of considering stage of lactation when interpreting genetic associations with energy intake and underscores the dynamic nature of intake regulation in pasture-based production systems.

In conclusion, the approximately linear increase in NEI with increasing genetic merit for live weight across all stages of lactation supports the continued use of live weight in total merit indexes as a means of shifting (or holding constant) a population in a given direction for energy intake, at least in Irish grazing Holstein-Friesian dairy cows. The potential to change NEI in a population can be improved by also having relevant (i.e., representative of the environment where the cows are producing) individual cow feed intake phenotypic data.
